# Age-related declines in mitochondrial Prdx6 contribute to dysregulated muscle bioenergetics

**DOI:** 10.1016/j.redox.2025.103808

**Published:** 2025-08-05

**Authors:** Jose Adan Arevalo, Dianna Xing, Roberto Garcia Leija, Max A. Thorwald, Diana Daniela Moreno-Santillán, Kaitlin N. Allen, Giovanna Selleghin-Veiga, Heidi C. Avalos, Eva Utke, Justin L. Conner, George A. Brooks, José Pablo Vázquez-Medina

**Affiliations:** a Department of Integrative Biology, University of California, Berkeley, USA; b Leonard Davis School of Gerontology, University of Southern California, USA

## Abstract

An age-related decline in mitochondrial function is a multi-factorial hallmark of aging, driven partly by increased lipid hydroperoxide levels that impair mitochondrial respiration in skeletal muscle, leading to atrophy. Although pharmacological and genetic manipulations to counteract increased lipid hydroperoxide levels represent a promising strategy to treat sarcopenia, the mechanisms driving such phenotypes remain understudied. Peroxiredoxin 6 (Prdx6) is a multifunctional enzyme that contributes to peroxidized membrane repair via its phospholipid hydroperoxidase and phospholipase A_2_ activities. Here, we show decreased mitochondrial Prdx6 levels, increased mitochondrial lipid peroxidation, and dysregulated muscle bioenergetics in aged mice and muscle cells derived from older humans. Mechanistically, we found that Prdx6 supports optimal mitochondrial function and prevents mitochondrial fragmentation by limiting mitochondrial lipid peroxidation via its membrane remodeling activities. Our results suggest that age-related declines in mitochondrial Prdx6 contribute to dysregulated muscle bioenergetics, thereby opening the door to therapeutic modulation of Prdx6 to counteract diminished mitochondrial function in aging.

## Graphical abstract

**Figure:**
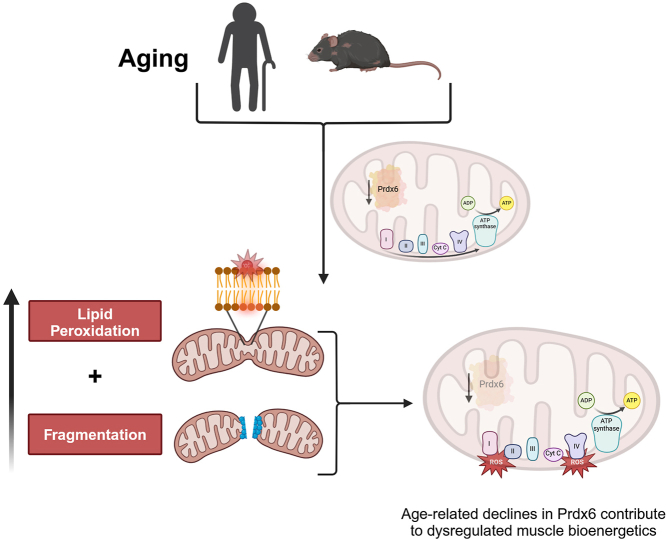

## 1 Introduction

An age-related decline in skeletal muscle mitochondrial function was first documented over two decades ago, and mitochondrial dysfunction is now recognized as a hallmark of aging [[1], [2], [3], [4], [5]]. Diminished mitochondrial function in aging is attributed to increased mitochondrial oxidant generation [[6], [7], [8]], decreased ability to induce antioxidant gene expression [9], and dysregulated mitophagy and mitochondrial dynamics [10,11], which lead to increased fragmentation [12] and decreased mitochondrial content [13,14]. Decreased mitochondrial function in aging precedes the loss of muscle mass and function [[15], [16], [17]], whereas mitochondrial fragmentation predicts age-associated declines in physical capacity [18]. Hence, understanding the factors driving alterations in aging muscle mitochondria is essential for developing strategies to mitigate age-related muscle atrophy.

Previous work shows increased lipid hydroperoxide generation in muscle mitochondria from young and aged mice and in models of accelerated aging [[19], [20], [21], [22]]. Furthermore, genetic or pharmacological modulation of lipid hydroperoxide levels ameliorates age-related muscle atrophy [[23], [24], [25], [26]]. While these phenotypes have primarily been attributed to glutathione peroxidase 4 (GPx4), the role of other lipid hydroperoxidases in this process remains elusive. Peroxiredoxin 6 (Prdx6) is a multifunctional enzyme that can reduce lipid hydroperoxides [27,28] and replace *sn*-2 fatty acyl groups in oxidized phospholipids through hydrolysis/reacylation via its phospholipase A_2_ (PLA_2_) and lysophosphatidylcholine acyl transferase activities [[29], [30], [31], [32]]. Cells lacking Prdx6 show increased lipid peroxidation [33,34], and overexpression of wildtype Prdx6 rescues this phenotype; however, overexpression of mutant Prdx6 with either peroxidase or PLA_2_ activity alone shows partial effect [33]. Similarly, genetic inactivation of the distinct Prdx6 activities via single-point mutations results in incomplete lipid peroxidation repair [35]. In contrast, the mutation that impairs Prdx6's ability to bind to phospholipids emulates the knockout phenotype, suggesting that both the peroxidase and phospholipid hydroperoxidase activities of Prdx6 are crucial to suppress lipid peroxidation [28,36,37]. The role of Prdx6 in aging skeletal muscles remains understudied. Still, previous work demonstrates that Prdx6 deficiency induces a sarcopenic-like phenotype in mice [38], and age-related Prdx6 dysregulation in myogenic progenitor cells increases mitochondrial oxidant generation [39]. Although Prdx6 was not originally associated with mitochondria, earlier work shows Prdx6 translocates to mitochondria during oxidant stress [40,41], and Prdx6 deletion induces mitochondrial dysfunction [[42], [43], [44]]. However, the mechanism by which Prdx6's activities support mitochondrial function and whether mitochondrial Prdx6 declines with aging remains unknown.

Here, we show decreased mitochondrial Prdx6 levels, increased mitochondrial lipid peroxidation, and disrupted mitochondrial architecture and bioenergetic profiles in skeletal muscle from aged mice and primary muscle cells derived from old humans. Using mice lacking Prdx6 or harboring single-point mutations that target the distinct Prdx6 activities, as well as Prdx6-depleted young human muscle cells, we demonstrate that Prdx6 supports optimal mitochondrial function by limiting mitochondrial lipid peroxidation and preserving mitochondrial architecture. Hence, our data suggest that age-related declines in mitochondrial Prdx6 contribute to dysregulated muscle bioenergetics.

## 2 Methods

### 2.1 Animals

Animal work was approved by the UC Berkeley Animal Care and Use Committee. Young (3–5 months) and aged (21–24 months) male and female WT C57BL/6 mice, and young Prdx6 null (KO), or knock-in (KI) mice harboring different Prdx6 mutations in the C57BL/6 background were used. The generation of Prdx6 KO mice is described in Ref. [45]. The generation of Prdx6 KI mice [46] is described in Refs. [32,35]. Prdx6-D140A mice harbor a single amino acid mutation at D140, one of the constituents of the Prdx6-PLA_2_ catalytic triad. This mutation abolishes the PLA_2_ activity without affecting the peroxidase activity. Prdx6-C47S mice lack Prdx6 peroxidase activity due to a single-point mutation in the catalytic cysteine (C47) but retain the PLA_2_ activity. The Prdx6-H26A mutation abolishes the ability of Prdx6 to hydrolyze or reduce oxidized phospholipids while preserving the peroxidase activity towards H_2_O_2_ and short-chain hydroperoxides, as this mutation prevents Prdx6 from binding to phospholipid substrate, but keeps the C47 residue intact [28,35,47]. The neomycin resistance cassette used in clonal selection during the generation of the Prdx6 KI mice was removed using flanking flippase recombinase target sites. Unaltered Prdx6 protein levels in KI mice have been previously confirmed [36,48]. Prdx6 KO and KI mice were generated originally by Dr. Aron Fisher (University of Pennsylvania), obtained through the Mutant Mouse Resource and Research Center (MMRRC), bred, and maintained in our facilities. Mixed muscles (gastrocnemius and quadriceps) from the hindlimb were isolated from both mouse legs. One set of muscles was placed in mitochondrial isolation buffer and used immediately for high-resolution respirometry. The other set was flash-frozen in liquid nitrogen and stored at −80 °C until analysis.

### 2.2 Preparation of mitochondrial fractions

Tissues were dissected as previously described [49], weighed, placed in ice-cold mitochondrial isolation buffer (250 mM mannitol, 10 mM EDTA,45 mM Tris-HCI, 5 mM Tris base, 0.15 % protease inhibitor, pH 7.4), minced, and homogenized using a Potter-Elvehjem homogenizer with a Teflon pestle. Mitochondria were isolated by differential centrifugation at 4 °C. The homogenate was centrifuged at 600*×g* for 10 min to pellet debris and nuclei. The supernatant was transferred to a second tube and centrifuged at 10,000*×g* for 10 min to pellet the mitochondrial fraction. Mitochondria were resuspended in isolation buffer (0.5 μL/mg of initial muscle weight) and kept on ice. Protein concentrations were determined using a Rapid Gold BCA kit (Thermo Fisher, Waltham, MA). Enrichment of the mitochondrial fractions was evaluated by Western blot.

### 2.3 High-resolution respirometry and H_2_O_2_ generation

Mitochondrial oxygen consumption and hydrogen peroxide (H_2_O_2_) generation were measured using a high-resolution FluoRespirometer (O2k, OROBOROS Instruments, Innsbruck, Austria). The respiration chamber was set at 37 °C, filled with 2 mL of MiR05 + DTPA (110 mM sucrose, 60 mM K-lactobionate, 0.5 mM EGTA, 3 mM MgCl2, 20 mM taurine, 10 mM KH_2_PO_4_, 20 mM HEPES, 0.1 % fatty acid-free BSA, pH 7.1) and continuously stirred. Following a period of equilibration, the chamber was closed, and the following additions were made to measure H_2_O_2_ generation: 10 μM Amplex UltraRed, 1 U/mL horseradish peroxidase, and 5 U/mL superoxide dismutase, followed by another equilibration period. Subsequently, 80 μg of mitochondria were added to initiate a modified substrate-uncoupler-inhibitor titration protocol. Pyruvate (10 mM) and malate (2 mM) were added to obtain state 2 respiration, followed by sub-saturating amounts of ADP to a final concentration of 50 μM to obtain state 3 and 4 respiration through complex I (CI), which was used to calculate Respiratory Control (RCR) and ADP to oxygen (P:O) ratios. After that, saturating amounts of ADP were added at 2.5 mM final concentration, followed by succinate (10 mM) to obtain maximal flux rates through CI and complex II (CII). This was followed by oligomycin addition (2 μg/mL) to obtain the rate of LEAK respiration (S4 omy) and calculate the percentage of proton leak through the inner mitochondrial membrane. Finally, titrations of FCCP (0.5–1 μM) were used to determine the uncoupled rate of respiration before the addition of antimycin A (2.5 μM) to inhibit complex lll (CIII) and measure residual oxygen consumption (ROX). Titrations of H_2_O_2_ (0.1 μM) were added following titrations of mitochondria, oligomycin, and antimycin A to account for the loss in sensitivity of Amplex UltraRed throughout the protocol.

### 2.4 Respirometry in frozen samples

Mitochondrial complex activities were measured using the respirometry in frozen samples (RIFS) protocol [50,51]. Muscle homogenates were prepared in mitochondrial isolation buffer. Total protein content was measured using a Rapid Gold BCA kit. Samples were diluted in MAS buffer (70 mM sucrose, 220 mM D-mannitol, 10 mM KH2PO4, 5 mM MgCl_2_, 2 mM HEPES, 1 mM EGTA, pH 7.2). 10 μg of protein were loaded onto Seahorse V3-PS XF24 microplates (Agilent Technologies, Santa Clara, CA). Plates were centrifuged at 2000*×g* for 20 min without brake at 4 °C. MAS buffer with cytochrome *c* (10 μg/ml) was added to bring the volume to 500 μL. Plates were equilibrated in a non-CO_2_ incubator (5–10 min, 37 °C) before assaying oxygen consumption in an XF24-3 extracellular flux analyzer (Agilent). The following substrates were added to measure complex activities: 1 mM NADH, 5 mM pyruvate, and 5 mM malate for CI, followed by rotenone with antimycin A (2 μM and 4 μM, respectively) to inhibit CI and CIII. For complex IV (CIV), 1 mM ascorbate and 0.5 mM N,N,N′,N'-tetramethyl-p-phenylenediamine (TMPD) were injected, followed by azide (20 mM). The instrument running protocol was modified to perform measurement intervals with 30-sec mix and 4-min intervals.

### 2.5 Mitochondrial lipid peroxidation and enzyme activities

The lipid peroxidation product malondialdehyde (MDA) was measured in isolated mitochondria using an MDA Adduct Competitive ELISA kit (Cell Biolabs, San Diego, CA, catalog no. STA-832). Total glutathione (GSH) peroxidase activity was measured using a commercial assay (Cayman Chemical, Ann Arbor, MI, catalog no. 703102) and normalized to total protein content measured using a BCA assay. Phospholipid hydroperoxide GSH peroxidase activity was measured in whole muscle extracts and mitochondrial fractions by substituting cumene hydroperoxide with 1-palmitoyl-2-linoleoyl-sn-glycero-3-phosphocholine hydroperoxide (PLPCOOH). PLPCOOH was prepared as previously described [52] following [53,54]. Briefly, 5 mg of L-α-phosphatidylcholine Type III/S (Sigma, St. Louis, MO, catalog no. P3782) was oxidized using 250,000 units of soybean lipoxidase Type V (Sigma, catalog no. L6632) for 1 h in 20 mL of 200 mM Tris 3 mM sodium deoxycholate pH 8.8. Oxidized lipids were collected using a Sep-Pak C18 cartridge (Waters, Milford, MA, catalog no. WAT 022515), washed, and eluted in methanol. PLPCOOH concentration was determined by measuring absorbance at 234 nm, using the extinction coefficient of 25,000 M^−1^cm^−1^. Assays were run in triplicate at 37 °C with 75 μg of lysate and 30 μM of PLPCOOH. Activity was calculated using the extinction coefficient for NADPH at 340 nm. Total protein content was measured using a BCA assay.

### 2.6 Western blot

Immunoblotting was conducted as described previously [55]. Briefly, 20–40 μg of protein prepared from mitochondrial fractions or whole muscle homogenates were diluted in LDS sample buffer containing reducing reagent and incubated for 10 min at 70 °C. Samples were run in BT gels, transferred onto PVDF or nitrocellulose membranes, probed with Revert 700 total protein stain solution (LI-COR Biosciences, Lincoln, NE, catalog no. 926–11011), imaged using a two-color NIR system (Azure c500 or Azure 500, Azure Biosystems, Dublin, CA), blocked using commercial (TBS) protein-free blocking buffer (LI-COR), and incubated overnight with primary antibodies. The next day, the membranes were washed and incubated with secondary antibodies (LI-COR IRDye 800CW) diluted 1:5000 to 1:10,000, and then imaged. Primary antibodies were used at the following dilutions: Prdx6 1:1000 (Cell Signaling Technologies, Danvers, MA, catalog no. 95336S), GPx4 1:2000 (Abcam, Cambridge, MA, catalog no. ab125066), Prdx3 1:1000 (Proteintech, Rosemont, IL, catalog no. 10664-1-AP), COXIV 1:2000 (Cell Signaling Technologies catalog no. 4844S), GAPDH 1:5000 (Cell Signaling Technologies catalog no. 2118), α-tubulin 1:1000 (Cell Signaling Technologies catalog no.2144S), and CPT2 1:1000 (Abcam no. ab181114). Densitometry was conducted using ImageJ. Data comparing young and aged animals were normalized to total protein, since using housekeeping proteins is uniquely vulnerable to biological variability [56]. This previously validated approach is recommended for complex tissues [[57], [58], [59], [60], [61], [62]]. For Prdx6, we confirmed our results by normalizing with an endogenous mitochondrial protein (CPT2) (Fig. S1C).

### 2.7 Cell models

Primary human skeletal muscle cells derived from young (19.67 ± 1.70 years) and older (67.0 ± 1.41 years) healthy, non-obese males (N = 3 per age group) were obtained from Cook Myosite (Pittsburgh, PA). Cells were isolated from the rectus abdominis and maintained in complete growth media consisting of Ham's F-10 nutrient mix (Gibco, Thermo Fisher) supplemented with 20 % fetal bovine serum (Seradigm, VWR, Radnor, PA), 10 mM HEPES (Gibco), and 1X Antimycotic/Antibiotic solution (Gibco). All experiments were conducted in cells between passages 5 and 7. C2C12 mouse myoblasts were obtained from ATCC through the UC Berkeley Cell Culture Facility and maintained in complete growth media supplemented with FBS and antibiotics. C2C12 cells stably expressing Cas9 were purchased from GeneCopoeia, Inc. (Rockville, MD, catalog no. SL564) and maintained in DMEM supplemented with FBS and 200 μg/mL hygromycin.

### 2.8 CRISPR/Cas9-mediated gene knockout and gene silencing

Prdx6 KO C2C12 cells were generated using the CRISPR/Cas9 system. Prdx6 expression was knocked down (KD) in primary skeletal muscle cells derived from young humans using siRNAs, as described in our previous work [43]. gRNAs targeting Prdx6 and non-targeting (NT) control sequences were obtained from IDT (Integrated DNA Technologies, Coralville, Iowa, catalog no. 416700882 and 1072544). Silencer-select pre-designed siRNAs targeting Prdx6 and nontargeting (NT) sequences were obtained from Invitrogen (Carlsbad, CA, catalog no. 4390824, assay ID s18429, catalog no. 4390844). Cells were transfected with 200 pmol of gRNAs (Cas9-expressing C2C12 cells) or 400 pmol of siRNAs (primary human skeletal muscle cells) using Lipofectamine RNAiMAX (Invitrogen, catalog no. 13778150). Prdx6 KO or KD was confirmed by Western blot 72-h post-transfection.

### 2.9 RNA-seq

Total RNA was extracted using an RNeasy Plus Kit (Qiagen, Redwood City, CA, catalog number: 74136) and quantified with a Nanodrop spectrophotometer (Thermo Fisher) and a Qubit fluorometer (Invitrogen). RNA integrity was measured using an Agilent 2100 Bioanalyzer. cDNA libraries were prepared from poly(A)-captured mRNA and sequenced to 20 million reads per sample on an Illumina platform (Illumina, San Diego, CA). Raw reads were filtered with Trim Galore to remove adapters, and low-quality reads [63]. Filtered reads were mapped to the human genome (version GRCh38.111) using STAR aligner [64]. Transcripts were quantified using RSEM [65]. Genes differentially expressed (DE) between cells transfected with NT and Prdx6 siRNAs were identified in EBSeq using a Bayesian approach at an FDR of 5 % [66]. Gene set enrichment analysis [67] with Gene Ontology (GO) was used to identify biological processes enriched in Prdx6-depleted cells.

### 2.10 qRT-PCR

cDNA was synthesized from RNA using a High-Capacity cDNA Reverse Transcription kit (Invitrogen catalog number: 4368814). qRT-PCR was performed with DyNAmo Flash SYBR Green master mix (Thermo Fisher catalog number: A25742) under the following conditions: 2 min at 50 °C, 10 min at 95 °C, followed by 40 cycles of 15 s at 95 °C and 1 min at 60 °C, and finishing at 95 °C. Relative expression of Prdx6 mRNA was calculated using the comparative 2^−ΔΔCt^ method using the geometric mean of *gapdh* and *actin* as housekeeping genes [68]. Primer sequences are listed in Table S1.

### 2.11 Measurement of mitochondrial function in intact cells

Cells seeded overnight in Seahorse mini plates were washed with XF assay medium (Seahorse XF DMEM, pH 7.4) and equilibrated in a CO_2_-free incubator for 1 h at 37 °C. Human primary skeletal muscle cells were seeded at 40,000 cells per well. Cas9-expressing C2C12 cells were seeded at 20,000 cells per well. Oxygen consumption rates (OCR) were measured in a Seahorse XFp extracellular flux analyzer. A stable baseline was measured for 20–30 min (3 measurements) before subsequent injections of oligomycin (1 μM), carbonyl cyanide-p-trifluoromethoxyphenylhydrazone (FCCP, 2 μM), and rotenone/antimycin A (0.5 μM). Non-mitochondrial OCR was subtracted from all values of mitochondrial function to determine basal OCR, ATP-linked OCR, proton leak, maximal OCR, and reserve capacity [69]. OCR was measured in cells derived from three young and three old individuals, and three independent transfections with control or Prdx6 gRNAs.

### 2.12 Detection of mitochondrial lipid peroxides in live cells

Cells seeded in collagen-coated glass bottom dishes (Ibidi, Fitchburg, WI) were labeled with 100 nM MitoTracker Red-CMXRos (Cell Signaling Technology, catalog no. 9082) and NucBlue (Thermo Fisher catalog no. R37605), and loaded with the mitochondrially-targeted lipid hydroperoxide probe MitoPeDPP [70] (0.5 μM, Dojindo, Rockville, MD, catalog no. M466) for 15 min. Cells were imaged on a Zeiss Axio Observer inverted microscope fitted with a 20× objective and Zen software. Following initial image acquisition, cells were treated with 200 μM *tert*-butyl hydroperoxide (tBOOH, Thermo Fisher catalog no. A13926) and imaged 30 min and 1 h after treatment. MitoPeDPP fluorescence intensity was measured in individual cells using ImageJ and normalized to vehicle-treated cells to calculate fold change. At least six cells from different fields within each independent sample were captured and analyzed.

### 2.13 Detection of mitochondrial oxidant generation

Mitochondrial oxidant generation was measured in C2C12 cells with and without Prdx6 KO. Cells seeded in collagen-coated glass-bottom dishes (Ibidi) were loaded with 50 nM MitoTracker Red CM-H2XRos (Thermo Fisher, catalog no. M7513). MitoTracker Red CM-H_2_XRos is a reduced mitochondrial dye that does not fluoresce until it is oxidized. Cells were imaged in live cell imaging solution (Invitrogen, catalog no. A59688DJ) using a Zeiss Axio Observer inverted microscope fitted with a 20× objective and Zen software. Fluorescence intensity was quantified using ImageJ in at least five different fields captured from three independent samples per group.

### 2.14 Lattice light-sheet microscopy with SIM^2^ 3D image reconstruction

Cells seeded in glass-bottom dishes were labeled with 100 nM MitoTracker Red-CMXRos and NucBlue, fixed, and imaged on a Zeiss Elyra 7 Lattice SIM2 epifluorescence super-resolution microscope equipped with a 63× oil objective. Z-stacks of 20–30 slices per cell were analyzed using Imaris software (Oxford Instruments, Pleasanton, CA, version 10.1). Surfaces were added to the mitochondrial image to measure volume (the space a surface object occupies) and disconnected components (the number of disconnected surfaces). At least five different cells per independent sample were imaged and used for analysis.

### 2.15 Immunofluorescence

Immunofluorescence was conducted following our previously published methods [48,55]. Briefly, cells grown in glass-bottom plates were stained with NucBlue and MitoTracker Red, fixed, permeabilized, and incubated overnight with anti-Prdx6 antibodies diluted 1:200. Prdx6 antibodies were a kind gift from Dr. Aron Fisher (University of Pennsylvania). Prdx6 antibodies are described in Refs. [71,72] and were previously validated using Prdx6 KO tissues and cells [43,48]. Alexa Fluor Plus 488 secondary antibodies (catalog no. A32731) were used at a 1:200 dilution. Cells were imaged using a Zeiss Axio Observer fluorescence microscope fitted with a 63× objective and Zen software. The relative abundance of Prdx6 in the mitochondrial and nuclear compartments was estimated by measuring the co-localization of the green (Prdx6) with the red (mitochondria) and blue (nucleus) channels in ImageJ. The relative cytosolic abundance of Prdx6 was calculated by subtracting mitochondrial and nuclear abundance from the total cell signal.

### 2.16 Duolink in situ proximity ligation

Visualization of protein-protein co-localization in intact cells was conducted using Duolink II in situ proximity ligation (Olink, Uppsala, Sweden) with the following antibodies: rabbit anti-Prdx6 (Abcam, Cambridge, MA, catalog number: ab125066), mouse anti-Prdx6 (EMD Millipore, Burlington, MA, catalog no. MABN1797), mouse anti-SOD2 (Invitrogen, catalog no. MA1-106), and mouse anti-TOM20 (Proteintech, catalog no. 66777-1-Ig), following the procedure described previously [43,55]. Nonspecific mouse and rabbit IgGs were used as negative controls. Cells were fixed with 1:1 ice-cold methanol/acetone, washed, permeabilized, treated with blocking reagent for 1 h, and incubated overnight with primary antibodies diluted 1:100. The Duolink II kit contains secondary antibodies against rabbit and mouse IgG, each attached to a unique synthetic oligonucleotide; if the two proteins are in proximity, ligation causes the two oligonucleotides to hybridize, allowing DNA replication and amplification of a red fluorescent signal [73,74]. The resulting signal was imaged with a Zeiss Axio Observer fluorescence microscope fitted with a 63× objective and Zen software.

### 2.17 Structural data

The structure of the mouse Prdx6 protein, highlighting the functional residues involved in the enzyme's catalytic activities, and mutated in the mouse KI strains, was retrieved using ChimeraX (https://www.cgl.ucsf.edu/chimerax/).

### 2.18 Statistical analysis

Statistical analyses were performed using GraphPad Prism 10.2.0 software (GraphPad Software Inc, Boston, MA). Comparisons between two groups were conducted using *t*-tests. Comparisons among several groups were conducted using ANOVA with Tukey post hoc analysis. Parametric tests were chosen after confirming the data's normality. Statistical significance was set at *α = 0.05*. Data are expressed as mean ± SD.

## 3 Results

### 3.1 Decreased skeletal muscle mitochondrial peroxiredoxins, increased lipid peroxidation, and dysregulated mitochondrial function in aged mice

**Fig. 1:**
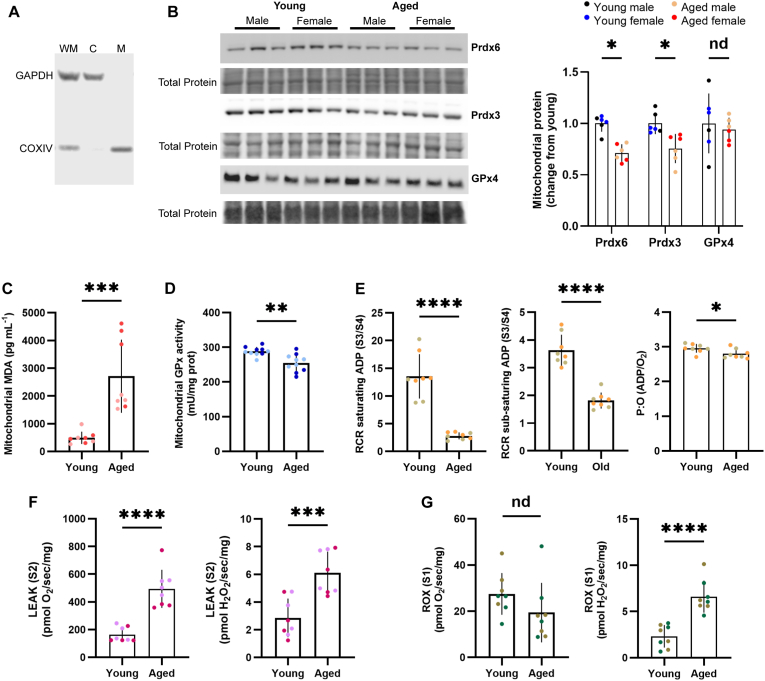
**Mitochondrial peroxiredoxins, lipid peroxidation, and bioenergetic profiles in skeletal muscles from young and aged mice.** A) Western blot for COXIV and GAPDH in whole muscle (WM), cytosolic (C), and mitochondrial (M) fractions. B) Prdx6, Prdx3, and GPx4 levels in mitochondrial fractions prepared from young and aged mouse muscle. C) Malondialdehyde (MDA) and D) Total GSH peroxidase activity in mitochondrial fractions. E) Respiratory control ratio under saturating and sub-saturating ADP conditions and P:O ratio under sub-saturating conditions in isolated mitochondria. LEAK (F) and ROX (G) respiration and simultaneous H_2_O_2_ generation. Data are mean ± SD. Different colors denote different sexes. ∗p < 0.05, ∗∗p < 0.01, ∗∗∗p < 0.001, ∗∗∗∗p < 0.001, nd = not significantly different.

We measured the levels of the prototypical mitochondrial peroxiredoxin (Prdx3), Prdx6, and GPx4 in skeletal muscle mitochondrial fractions prepared from male and female young and aged mice (Fig. 1A). While Prdx3 and Prdx6 levels were lower in aged animals than in young animals, GPx4 was not statistically different between age groups (Fig. 1B). In contrast, neither Prdx6 nor GPx4 levels were significantly different between age groups in whole muscle homogenates (Fig. S1A) or cytosolic fractions, although we observed a nonsignificant increase in cytosolic Prdx6 in aged animals (Fig. S1B). We then measured the lipid peroxidation product malondialdehyde (MDA) and total GSH peroxidase (GPx) activity in mitochondrial fractions. Consistent with the lower Prdx3 and Prdx6 levels in aged animals than in young animals, we found higher mitochondrial MDA (Fig. 1C) and lower mitochondrial GPx activity in aged mice (Fig. 1D). Both Prdx3 and Prdx6 can reduce short-chain hydroperoxides and hydrogen peroxide [75,76], with Prdx3 acting as the primary hydrogen peroxide removal system in the mitochondrial matrix [77,78]. In contrast, Prdx6 is the only peroxiredoxin with reported non-selenium GSH phospholipid hydroperoxidase activity [27,36,[79], [80], [81]]. These results suggest that aging impairs mitochondrial hydroperoxide removal in skeletal muscle, coinciding with a decline in mitochondrial peroxiredoxins. We then conducted high-resolution respirometry in muscle mitochondria isolated from young and aged animals to test whether reduced mitochondrial peroxiredoxin levels correlate with dysregulated mitochondrial function. Consistent with previous reports [82,83], we found lower RCR and P:O ratios in aged than in young mice (Fig. 1E). Furthermore, mitochondria from aged animals exhibited higher LEAK respiration, lower ROX respiration, and higher H_2_O_2_ generation (Fig. 1F and G), suggesting that lower mitochondrial peroxiredoxin levels are associated with alterations in muscle bioenergetics.

### 3.2 Prdx6 deficiency suppresses mitochondrial function, increases H_2_O_2_ generation, and disrupts mitochondrial membrane integrity

**Fig. 2:**
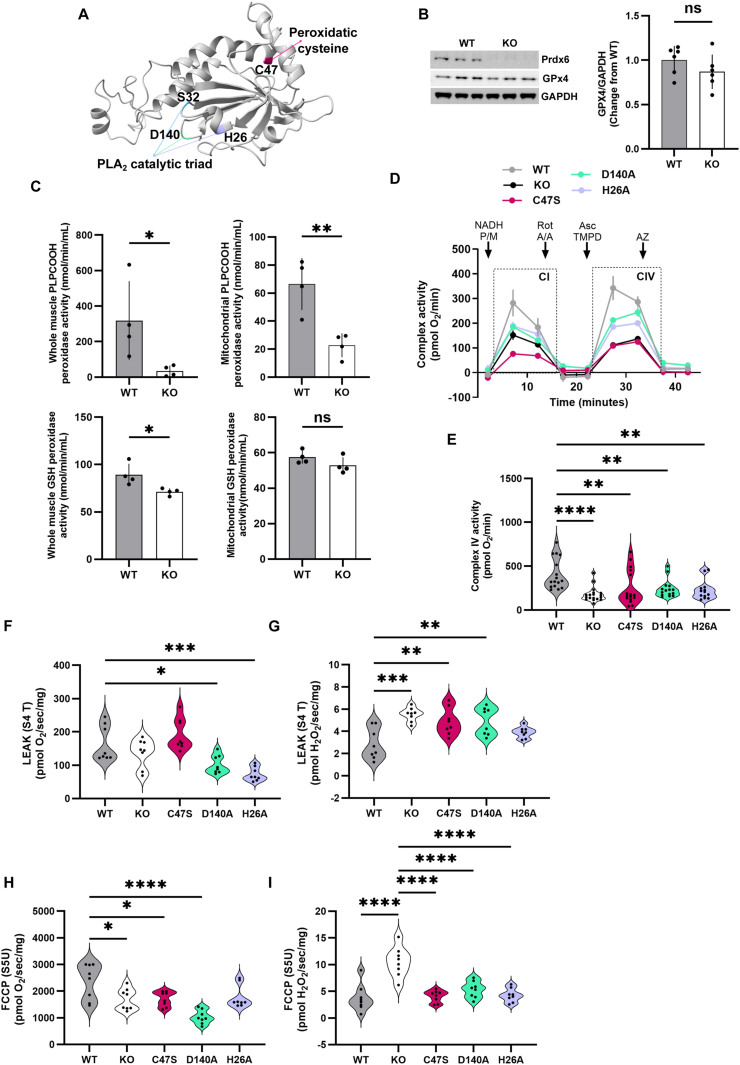
**Prdx6 deficiency impairs mitochondrial function.** A) Structure of mouse Prdx6, highlighting the functional residues involved in the catalytic activities of the protein. B) WB analysis for Prdx6 and GPx4 in skeletal muscle from young (3–5 months old) WT and Prdx6 KO mice. C) GSH peroxidase activity against PLPCOOH and cumene hydroperoxide in whole muscle and mitochondrial fractions. D) Representative experiment showing oxygen consumption during complex I (CI) and complex IV (CIV) activity measurements using the RIFS protocol in whole muscle from young WT, Prdx6 KO, and Prdx6 KI mice. P/M: pyruvate/malate; Rot: rotenone; A/A: antimycin A; Asc: ascorbate; AZ: azide. E) Complex IV activity in muscle from young WT, Prdx6 KO, and Prdx6 KI mice. F–I) High-resolution respirometry and H_2_O_2_ generation in skeletal muscle mitochondria. F, G) LEAK refers to State 4 (S4 T) CI-linked oxygen consumption without the addition of exogenous ADP. H, I) Maximal uncoupled respiration and simultaneous H_2_O_2_ generation after adding FCCP. ∗p < 0.05, ∗∗p < 0.01, ∗∗∗p < 0.001, ∗∗∗∗p < 0.0001, ns = not significantly different.

Prdx6 localizes primarily to the cytosol and lysosomal-type organelles under unstimulated conditions [84,85], and translocates to cell membranes where it exerts its PLA_2_ and phospholipid hydroperoxidase activities during oxidant stress and agonist stimulation [28,48,55,71,86]. Prdx6 was not originally associated with mitochondria, but new evidence shows that Prdx6 is a constituent of the mitochondrial proteome [87]. Furthermore, Prdx6 deletion dysregulates mitochondrial function [42,43]. Hence, we studied the effects of Prdx6 deficiency on mitochondrial function in skeletal muscles from young mice lacking Prdx6 or harboring single-point mutations that inactivate the peroxidase (C47S) or PLA_2_ (D140A) activities or suppress Prdx6's ability to bind to phospholipids (H26A; Fig. 2A). In contrast to recent work showing decreased GPx4 levels in Prdx6 KO cells [[88], [89], [90]], GPx4 levels were comparable in skeletal muscle from WT and Prdx6 KO mice (Fig. 2B), consistent with the previous observation that animals lacking Prdx6 have reduced GPx4 levels in some but not all tissues [89]. Notably, Phospholipid hydroperoxidase activity against PLPCOOH was blunted both in whole muscle and mitochondrial fractions from Prdx6 KO animals (by 90 % and 65 %, respectively, Fig. 2C), despite GPx4 being the primary enzyme that reduces phospholipid hydroperoxides [91,92]. These results suggest that Prdx6 contributes to limiting lipid peroxidation independently of GPx4 in skeletal muscle, similar to previous reports in SNU475 cells lacking Prdx6 [34]. Furthermore, GSH peroxidase activity against cumene hydroperoxide was only lower by 20 % in whole muscle from Prdx6 KO compared to WT mice (p = 0.0253, t = 2.959), and unaltered in mitochondrial fractions (Fig. 2C), further suggesting that lack of Prdx6 does not impact the levels of selenium-containing GPx enzymes in skeletal muscle. Thus, Prdx6 KO does not appear to affect GPx4 levels in skeletal muscle but significantly decreases phospholipid hydroperoxidase activity.

We first assessed whether respiratory chain complex activity varied in skeletal muscle from young WT, Prdx6 null, and Prdx6 KI mice using RIFS. CIV activity was 66 % lower in Prdx6 KO mice and 38–44 % lower in KI mice compared to WT (Fig. 2D and E). In contrast, CI activity did not differ between groups (Fig. S2), suggesting that Prdx6 primarily supports CIV activity. We then measured oxygen consumption and H_2_O_2_ generation in freshly prepared muscle mitochondria from young WT, Prdx6 KO, and Prdx6 KI mice to further investigate the role of the different catalytic activities of Prdx6 in supporting mitochondrial function. D140A and H26A mice showed lower basal mitochondrial oxygen consumption than WT mice, suggesting altered mitochondrial function due to a lack of Prdx6-PLA_2_ (Fig. 2F). Furthermore, KO, C47S, and D140A mice had significantly higher H_2_O_2_ generation rates during S2 respiration than WT (Fig. 2G). Combined with lower oxygen consumption, these results suggest damage to the ATP-generating machinery due to altered Prdx6. Additionally, maximal uncoupled respiration was significantly lower in KO, C47S, and D140A than in WT mice (Fig. 2H), whereas H_2_O_2_ generation was significantly higher in the KO than in all other strains, further suggesting that mitochondrial Prdx6 contributes to regulating mitochondrial redox balance (Fig. 2I).

**Fig. 3:**
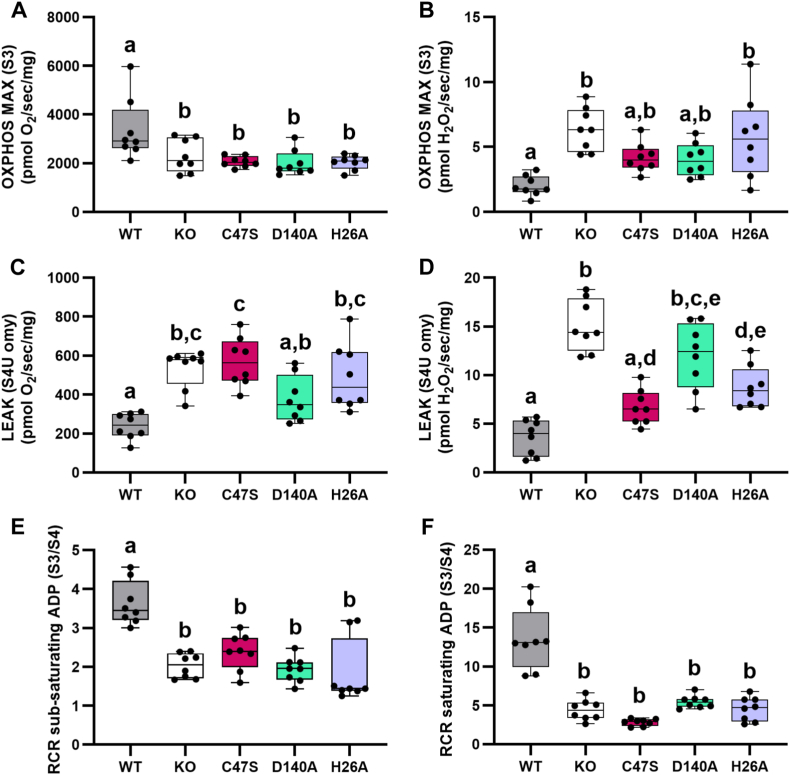
**Prdx6 maintains mitochondrial membrane integrity**. A) Maximal oxidative phosphorylation using saturating levels of ADP and B) simultaneous H_2_O_2_ generation measured using Amplex UltraRed. C) Oligomycin-induced state 4U (S4U omy) respiration predominantly measures proton leakage across the inner mitochondrial membrane. D) H_2_O_2_ generation in response to oligomycin inhibition of ATP synthase. Respiratory Control Ratio (RCR) calculated from S3 and S4 respiration using E) sub-saturating and F) saturating amounts of ADP. Different letters denote statistical differences among groups. All animals used for these experiments (WT, KO, and KI) were young (3–5 months old).

To further investigate the role of Prdx6 in maintaining mitochondrial membrane integrity, we measured the ratio of H_2_O_2_ generation to oxygen consumption during ADP-linked maximal respiration and proton leak in isolated mitochondria from young WT, Prdx6 KO, and Prdx6 KI mice. State 3 oxygen consumption was lower in KO and all KI strains than in WT mice (Fig. 3A). In contrast, H_2_O_2_ production was increased only in KO and H26A mice (Fig. 3B), suggesting potential damage to mitochondrial membranes and complexes due to inefficient removal of peroxidized phospholipids. H26A mice have an intact C47 residue, which is essential for peroxidase activity and the recently discovered function of Prdx6 as a selenium carrier [[88], [89], [90]], but Prdx6 in these animals cannot bind lipid membranes and therefore they lack both PLA_2_ and phospholipid hydroperoxidase activity [32,36,47]. Consistent with this observation, C47S and D140A mice, which can partially repair lipid peroxidation [35], did not show significant changes in H_2_O_2_ formation (Fig. 3B). Whereas both the peroxidase and PLA_2_ activities of Prdx6 participate in lipid peroxidation repair [33,35,43], previous work shows a complete absence of lipid peroxidation repair in primary pulmonary endothelial cells and lungs derived from H26A and Prdx6 KO mice [36]. Therefore, excessive H_2_O_2_ generation by KO and H26A mitochondria potentially reflects disruptions in mitochondrial membrane remodeling since the H26A mutation abolishes Prdx6's ability to bind phospholipids [47]. We also detected a higher oligomycin-linked proton leak in KO, C47S, and H26A mice, as well as increased H_2_O_2_ formation in KO, D140A, and H26A mice compared to WT mice, further suggesting that Prdx6 is crucial for supporting optimal ATP production (Fig. 3C and D). Lastly, RCRs derived from S3 and S4 respiration using sub-saturating and saturating amounts of ADP were significantly lower in all strains compared to WT (Fig. 3E and F), indicating overall mitochondrial dysfunction, potentially from a loss of mitochondrial membrane integrity and damage to mitochondrial complexes.

Skeletal muscle cells derived from old humans show reduced mitochondrial function, increased mitochondrial fragmentation, and lipid peroxidation.

**Fig. 4:**
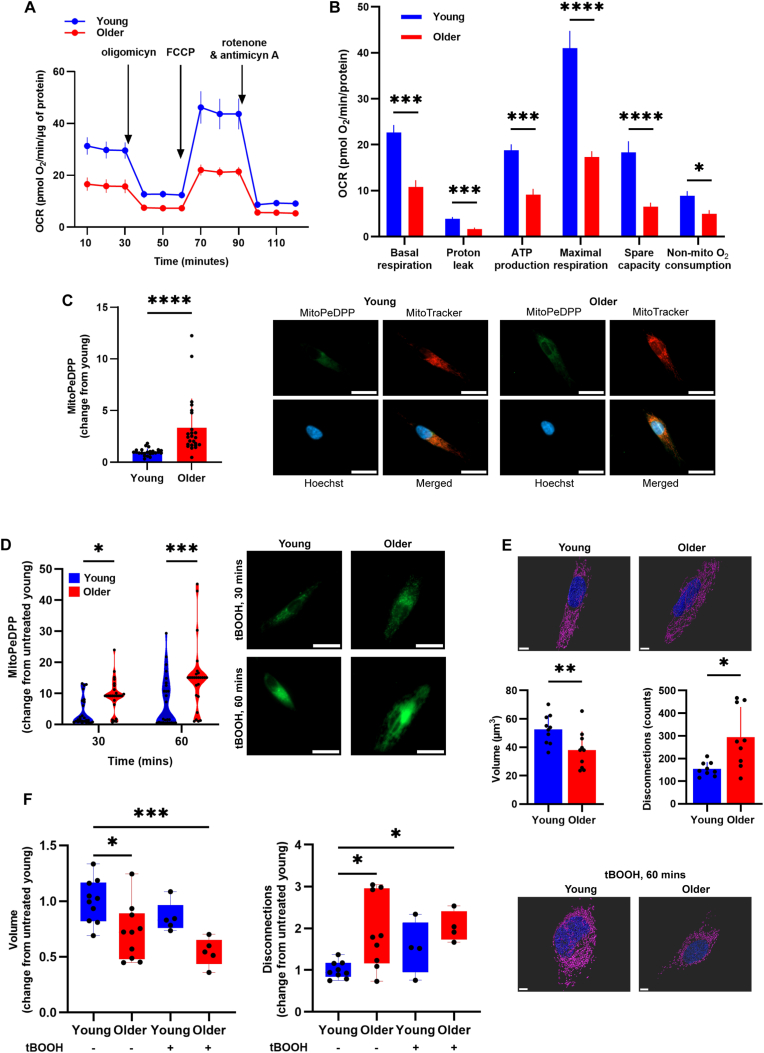
**Skeletal muscle cells derived from older humans exhibit decreased mitochondrial function, accompanied by increased mitochondrial fragmentation and lipid peroxidation.** A) Oxygen consumption rates (OCR) in primary skeletal muscle cells derived from young (19.67 ± 1.70 years) and older (67.0 ± 1.41 years) humans. B) Mitochondrial function calculated in (A) according to Ref. [69]. Data in A and B are mean ± SEM (N = 3 per group). C) Mitochondrial lipid peroxides measured using the mitochondrially-targeted lipid peroxide probe MitoPeDPP. D) Mitochondrial lipid peroxides in cells treated with 200 μM tBOOH. Scale bar is 25 μm. E) Analysis of the mitochondrial reticulum in cells imaged using Lattice light-sheet microscopy with SIM^2^ 3D image reconstruction. F) Analysis of the mitochondrial reticulum in cells exposed to tBOOH. Scale bar is 5 μm. Data are mean ± SD. ∗p < 0.05, ∗∗p < 0.01, ∗∗∗p < 0.001, ∗∗∗∗p < 0.0001.

Our data show that in mouse muscle, mitochondrial peroxiredoxins and mitochondrial function decline with age, in parallel with increases in mitochondrial lipid peroxidation. Similarly, previous work has demonstrated that dysregulated Prdx6 expression is associated with increased mitochondrial oxidant generation in skeletal muscle cells isolated from older humans [39]. Hence, we evaluated whether mitochondrial function and lipid hydroperoxide levels differ in intact primary skeletal muscle cells derived from young and older humans using extracellular flux assays and the mitochondrially targeted lipid peroxide probe MitoPeDPP [70]. Mitochondrial function was lower in cells isolated from older humans compared to young humans (Fig. 4A and B), whereas mitochondrial lipid peroxidation was higher in cells from older humans (Fig. 4C). Next, we tested whether mitochondrial lipid peroxidation in response to treatment with organic peroxides differs between cells derived from young and older humans. Lipid peroxidation was higher in cells from older than young humans in response to hydroperoxide treatment for 30 and 60 min (Fig. 4D). These results indicate that skeletal muscle cells from older humans exhibit lower mitochondrial function and a diminished capacity to scavenge mitochondrial lipid peroxides.

Next, we used Lattice light-sheet super-resolution microscopy with SIM^2^ 3D image reconstruction to study the three-dimensional architecture of the mitochondrial reticulum in primary muscle cells derived from young and older humans. We found that the volume of the mitochondrial network was lower in cells derived from older compared to young humans (young: 52.65 ± 10.27 vs older: 37.94 ± 13.23 μm^3^; Fig. 4E). Similarly, cells derived from older humans had more disconnected components (young: 153.6 ± 30.71 vs older: 293.5 ± 134.2 counts; Fig. 4E), suggesting increased mitochondrial fragmentation. We then studied the effect of hydroperoxide treatment on mitochondrial morphology (Fig. 4F). While disconnections in response to hydroperoxide increased in cells derived from both young and older humans, mitochondrial volume decreased only in cells from older humans. Thus, skeletal muscle cells derived from older humans have diminished mitochondrial function and increased mitochondrial fragmentation and lipid peroxidation compared to cells derived from young humans. Furthermore, cells from older humans are more susceptible to mitochondrial lipid peroxidation and fragmentation when exposed to hydroperoxides.

Peroxiredoxin 6 supports mitochondrial function and preserves mitochondrial architecture while suppressing mitochondrial lipid peroxidation.

**Fig. 5:**
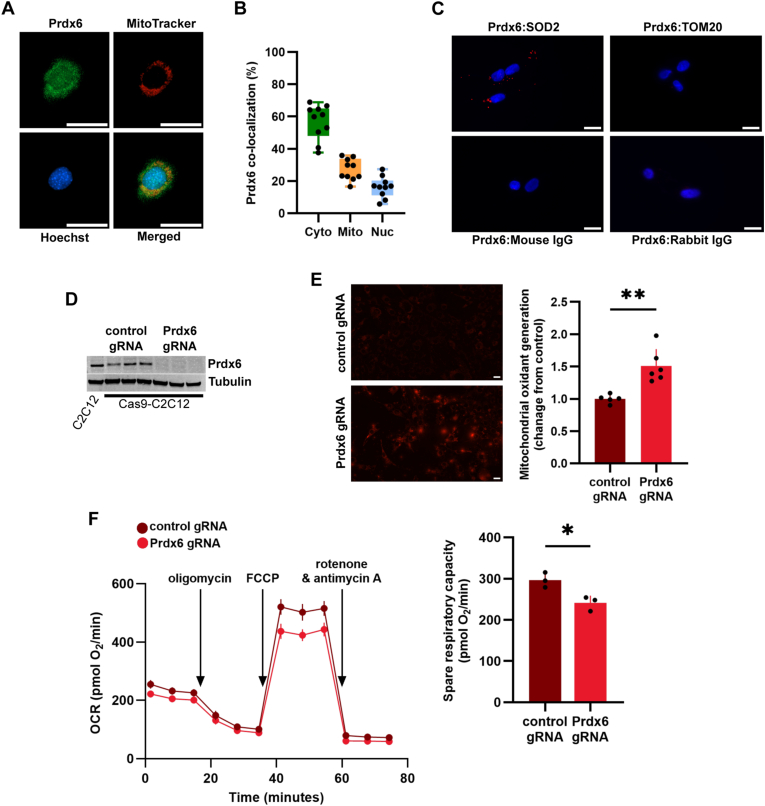
**Loss of Prdx6 impairs mitochondrial function.** A) Immunofluorescence for Prdx6 (green) in C2C12 cells stained with MitoTracker red and NucBlue. Scale bar is 25 μm. B) Relative abundance of Prdx6 in the cytosol, mitochondria, and nucleus of C2C12 cells. Prdx6 levels in each compartment were calculated by measuring Prdx6 co-localization (green channel) with the red (mitochondria) and blue (nucleus) channels. Cytosolic levels reflect the total signal for Prdx6 minus the mitochondrial and nuclear pools. C) Duolink in situ proximity ligation assay for Prdx6 co-localization with SOD2 and TOM20. Red dots indicate proximity (<40 nm) of the two proteins. The IgG panels represent the substitution of specific primary antibodies with rabbit and mouse IgGs (negative controls). Scale bar is 25 μm. D) Generation of Prdx6 KO C2C12 cells using CRISPR/Cas9. E) Mitochondrial oxidant generation measured using MitoTracker Red CM-H2XRos, a reduced mitochondrial dye that does not fluoresce until oxidized. Scale bar is 25 μm. F) Oxygen consumption rates and spare respiratory capacity in C2C12 cells with and without Prdx6 KO. ∗p < 0.05, ∗∗p < 0.01.

Using immunofluorescence analysis, we confirmed that Prdx6 localizes to mitochondria in C2C12 muscle cells (Fig. 5A), with 27 % of the protein present in mitochondria, 57 % in cytosol, and 16 % in the nucleus (Fig. 5B). We then used in situ proximity ligation to study whether Prdx6 colocalizes with known proteins in the mitochondrial matrix (SOD2) or the outer mitochondrial membrane (TOM20). We found associations between Prdx6 and SOD2, but not between Prdx6 and TOM20, suggesting that mitochondrial Prdx6 is primarily located in the mitochondrial matrix (Fig. 5C). We then generated Prdx6 KO C2C12 muscle cells (Fig. 5D) and tested whether loss of Prdx6 impacts mitochondrial function. Prdx6 KO increased mitochondrial oxidant generation (Fig. 5E) and blunted mitochondrial function, significantly decreasing spare respiratory capacity (Fig. 5F). These results further show that Prdx6 supports mitochondrial function in muscle cells.

**Fig. 6:**
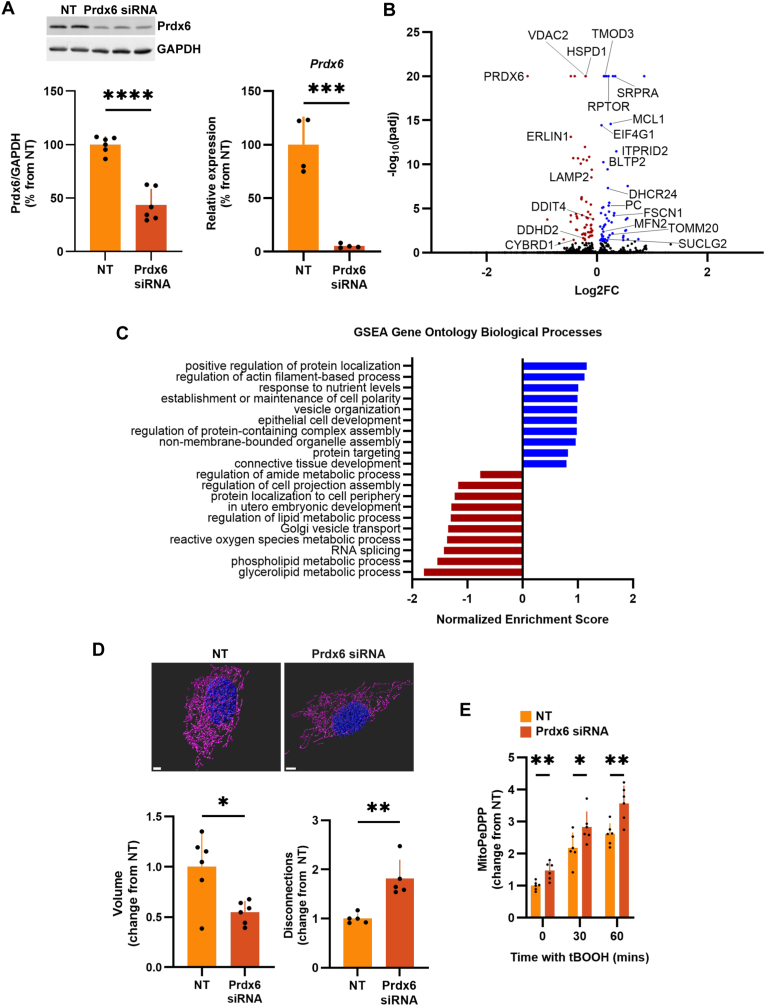
**Prdx6 supports mitochondrial function by suppressing mitochondrial lipid peroxidation and preserving mitochondrial integrity.** A) Prdx6 KD in skeletal muscle cells isolated from young humans. B) Effect of Prdx6 KD on differential gene expression. C) GSEA for GO biological processes enriched in Prdx6-depleted cells. In B) and C), blue represents upregulated genes and pathways, whereas red represents downregulated genes and pathways in cells transfected with Prdx6 siRNA vs NT siRNAs. D) Mitochondrial morphology in Prdx6-depleted cells measured using Lattice light-sheet microscopy with SIM^2^ 3D image reconstruction. Scale bar is 3 μm. E) Mitochondrial lipid peroxides measured using MitoPeDPP in cells treated with 200 μM tBOOH. ∗p < 0.05, ∗∗p < 0.01, ∗∗∗p < 0.001, ∗∗∗∗p < 0.0001.

We next investigated the role of Prdx6 in counteracting mitochondrial lipid peroxidation and maintaining mitochondrial reticulum morphology in primary skeletal muscle cells derived from young humans under basal conditions and following treatment with hydroperoxide. Prdx6 knockdown with siRNA reduced Prdx6 mRNA by 95 % and Prdx6 protein levels by 56 % (Fig. 6A). Prdx6-depleted cells showed differential expression of 59 upregulated and 56 downregulated genes under basal conditions. Top differentially expressed genes include several involved in muscle function, cellular stress, and energy metabolism (Fig. 6B), consistent with previous reports in C2C12 cells with Prdx6 KD and mixed muscles from Prdx6 KO animals [38]. Moreover, GSEA showed enrichment for GO biological processes involved in energy generation, oxidant stress, and lipid metabolism in Prdx6-depleted cells (Fig. 6C). Prdx6 depletion decreased mitochondrial volume while increasing mitochondrial disconnections (Fig. 6D). Furthermore, mitochondrial lipid peroxidation was higher under basal conditions and in response to hydroperoxide in Prdx6-depleted cells (Fig. 6E). Together, these results show that Prdx6 supports mitochondrial function by suppressing mitochondrial lipid peroxidation, which disrupts mitochondrial integrity. Overall, these results suggest that age-related declines in mitochondrial Prdx6 contribute to dysregulated mitochondrial function.

## 4 Discussion

Skeletal muscle mitochondrial dysfunction is a hallmark of aging that contributes to age-related muscle atrophy [5]. Increased lipid hydroperoxides and mitochondrial fragmentation promote mitochondrial dysfunction in aging, but the mechanisms driving such processes remain poorly understood [18,23,26]. Here, we show that mitochondrial Prdx3 and Prdx6 levels decrease with aging, in parallel with increases in mitochondrial lipid peroxidation and dysregulated muscle bioenergetics. Using Prdx6 knockout and mutant mice, as well as primary human muscle cells with depleted Prdx6 expression, we further demonstrate that both the PLA_2_ and phospholipid hydroperoxidase activities of Prdx6 support mitochondrial function by limiting mitochondrial lipid peroxidation and preserving mitochondrial architecture.

Prdx6 was not previously recognized as a mitochondria-associated protein, but emerging evidence, including work showing that Prdx6 deletion decreases mitochondrial function in lung and liver cells [[42], [43], [44],^87^], supports its inclusion as a constituent of the mitochondrial proteome [87]. Using subcellular fractionation, immunofluorescence, and in situ proximity ligation, we show that Prdx6 localizes to mitochondria in skeletal muscle. Furthermore, deletion of Prdx6 in muscle cells blunted mitochondrial function and increased mitochondrial oxidant generation, consistent with previous reports in myogenic precursor cells [39]. In contrast to the prototypical mitochondrial peroxiredoxin (Prdx3), Prdx6 does not contain a mitochondria-targeting sequence [93], but previous work shows that it can translocate to the mitochondrial matrix under oxidant stress [40,41,44,94]. Here, we show that Prdx6 is expressed in skeletal muscle mitochondria, and its levels decline with aging, coinciding with increases in mitochondrial lipid peroxidation and impaired bioenergetics. Moreover, ablation or mutation of Prdx6 impaired mitochondrial function, further demonstrating the role of Prdx6 in supporting optimal mitochondrial metabolism. Despite the obvious biological consequences of altered mitochondrial Prdx6 levels in skeletal muscle, mechanistic explanations regarding how Prdx6 localizes to mitochondria remain elusive. The use of GFP-SPLIT or mitochondrial sub-fractionation methods would help uncover the precise location of Prdx6 within the mitochondria.

Aging increases the accumulation of lipid peroxidation products that promote mitochondrial dysfunction, including isolevuglandins [95]. Whether age-related declines in mitochondrial Prdx6 are directly related to the accumulation of such products remains an open question that warrants further investigation. Our data show that primary muscle cells derived from older humans exhibit suppressed mitochondrial function, increased mitochondrial lipid peroxidation, and mitochondrial fragmentation under unstimulated conditions and upon exposure to hydroperoxides. Notably, Prdx6 depletion in cells derived from young humans mimics this phenotype, suggesting that Prdx6 is essential to suppress mitochondrial lipid peroxidation while maintaining mitochondrial architecture. Increased mitochondrial lipid peroxidation was recently linked to mitochondrial fragmentation [96,97], and previous work shows that Prdx6 expression increases in response to genetic or pharmacological perturbations of the mitochondrial reticulum in skeletal muscle myotubes [98]. Our studies in Prdx6 KO and KI animals suggest that both the phospholipid hydroperoxidase and PLA_2_ activities of Prdx6 likely contribute to its cytoprotective effect on mitochondrial function, consistent with previous observations that both Prdx6 activities contribute to limiting lipid peroxidation [28,33,[35], [36], [37],^43^]. Although we did not study aged Prdx6 KO or mutant mice, previous results suggest that Prdx6-deficient animals exhibit increased susceptibility to oxidant stress in aging [99], a sarcopenic-like phenotype [38], and potentially other aging-related metabolic diseases [39,[100], [101], [102], [103], [104]]. Furthermore, it is essential to note that while the observed declines in mitochondrial Prdx6 levels may contribute to dysregulated redox balance in aging, other pathways and factors that increase oxidant generation and suppress other mitochondrial antioxidant activities are well known [105,106]. Similarly, other mitochondrial thiol peroxidases, such as Prdx5 and GPx1, may also contribute to compensatory effects in the knockout and transgenic mouse strains.

In summary, we found decreased mitochondrial Prdx3 and Prdx6 levels, increased mitochondrial lipid peroxidation, and dysregulated bioenergetics in skeletal muscles from aged mice. Mechanistically, we found that Prdx6 supports optimal mitochondrial function and preserves mitochondrial architecture by suppressing lipid peroxidation via its membrane remodeling activities. Hence, age-related declines in Prdx6 likely contribute to dysregulated muscle bioenergetics in aging. Therefore, modulation of Prdx6 may be beneficial to counteract age-related muscle atrophy [38,100,107,108].

## CRediT authorship contribution statement

**Jose Adan Arevalo:** Writing – original draft, Visualization, Validation, Methodology, Investigation, Formal analysis, Data curation. **Dianna Xing:** Writing – original draft, Visualization, Validation, Methodology, Investigation, Formal analysis, Data curation. **Roberto Garcia Leija:** Writing – review & editing, Writing – original draft, Visualization, Validation, Methodology, Formal analysis, Data curation, Conceptualization. **Max A. Thorwald:** Writing – review & editing, Validation, Methodology, Investigation, Data curation. **Diana Daniela Moreno-Santillán:** Writing – review & editing, Validation, Investigation, Data curation. **Kaitlin N. Allen:** Writing – review & editing, Validation, Methodology. **Giovanna Selleghin-Veiga:** Writing – review & editing, Visualization, Software, Data curation. **Heidi C. Avalos:** Methodology, Data curation. **Eva Utke:** Investigation. **Justin L. Conner:** Investigation. **George A. Brooks:** Writing – review & editing, Resources, Methodology, Conceptualization. **José Pablo Vázquez-Medina:** Writing – review & editing, Writing – original draft, Visualization, Validation, Supervision, Resources, Project administration, Methodology, Investigation, Funding acquisition, Formal analysis, Data curation, Conceptualization.

## Declaration of competing interest

The authors declare that they have no known competing financial interests or personal relationships that could have appeared to influence the work reported in this paper.

## Data Availability

Data will be made available on request.
