# Supplementary material for: Age-related declines in mitochondrial Prdx6 contribute to dysregulated muscle bioenergetics
Source: Redox Biol. 2025 Aug 5;86:103808. doi: 10.1016/j.redox.2025.103808 (PMC12355596; doi:10.1016/j.redox.2025.103808)
Supplement: Multimedia component 1 [file mmc1.pdf]

Age-related declines in mitochondrial Prdx6 contribute to dysregulated muscle bioenergetics

Jose Adan Arevalo[1,], Dianna Xing[1,], Roberto Garcia Leija[1,], Max A. Thorwald[2], Diana Daniela Moreno-Santillán[1], Kaitlin N. Allen[1], Giovanna Selleghin-Veiga[1], Heidi C. Avalos[1], Eva Utke[1], Justin L. Conner[1], George A. Brooks[1] and José Pablo Vázquez-Medina[1,]

1. Department of Integrative Biology, University of California, Berkeley
2. Leonard Davis School of Gerontology, University of Southern California

Supplementary figures and tables

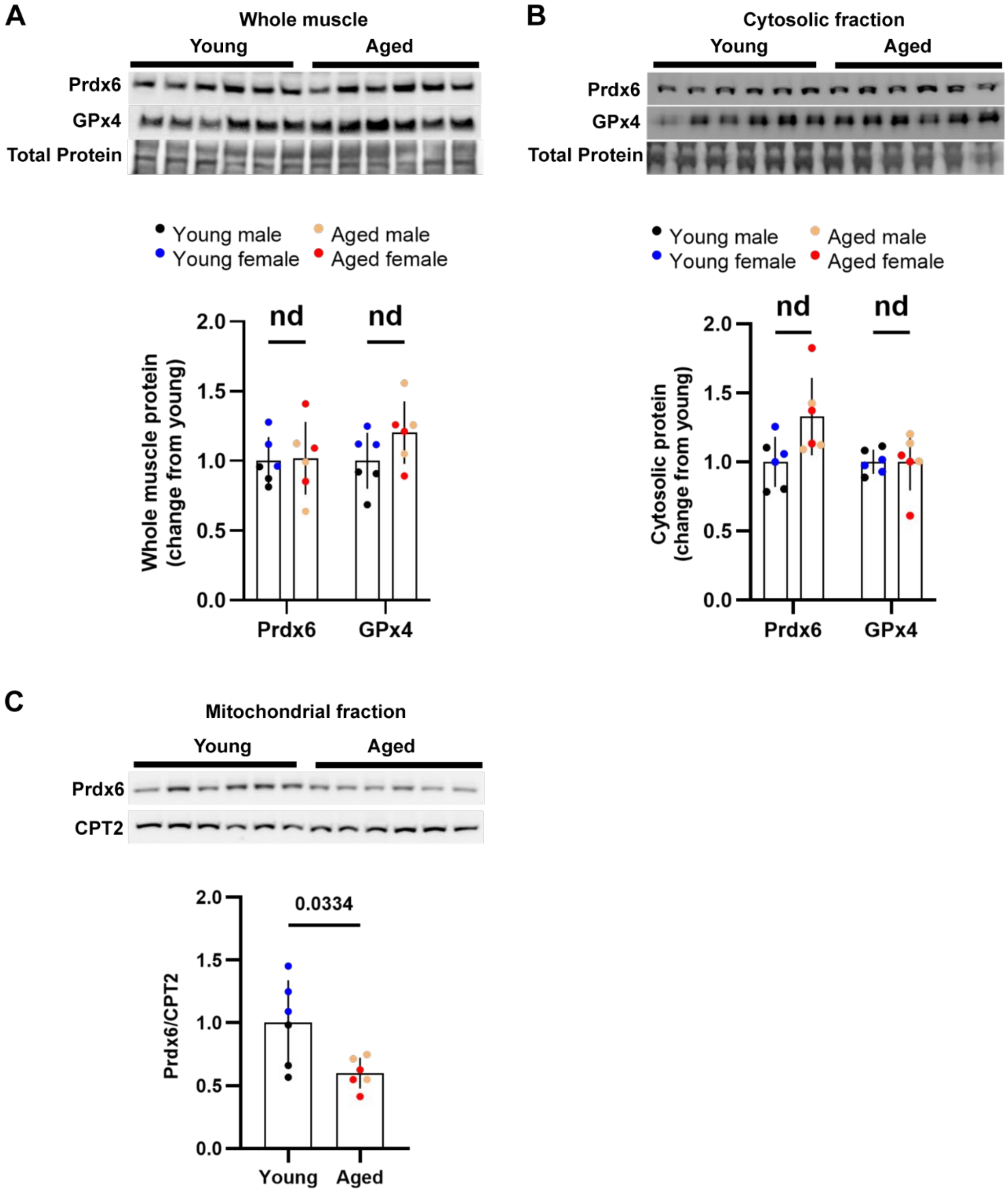

Figure S1. Mitochondrial Prdx6 levels decline with aging. WB analysis of Prdx6 and GPx4 in whole muscle (A), cytosolic (B), and mitochondrial fractions (C) prepared from young and aged mouse skeletal muscle. Data in C correspond to the data in Figure 1B, normalized to the endogenous mitochondrial protein CPT2.

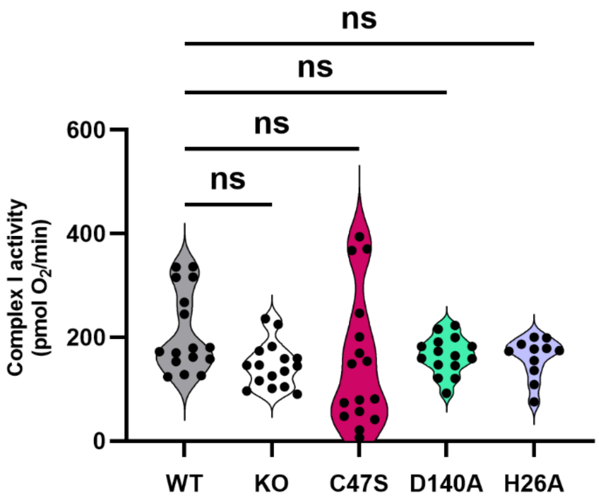

Figure S2. Prdx6 KO or KI does not impact mitochondrial complex I activity. Complex I activity in muscle homogenates from WT, Prdx6 KO, and Prdx6 KI mice measured using the RIFS Protocol.

Supplementary Table 1. Primer sequences

Gene	Sequence 5 ' to 3'
prdx6	CGT GTG GTG TTT GTT TTT GG<br>CCA TCA CAC TAT CCC CAT CC
gapdh	GGA TTT GGT CGT ATT GGG<br>GGA AGA TGG TGA TGG GAT T
Actin	CAC CAT TGG CAA TGA GCG GTT C<br>AGG TCT TTG CGG ATG TCC ACG T
